# Implementing Telemedicine in Clinical Practice in the First Digital Hematology Unit: Feasibility Study

**DOI:** 10.2196/48987

**Published:** 2023-12-04

**Authors:** Amparo Santamaria, Cristina Antón Maldonado, Beatriz Sánchez-Quiñones, Nataly Ibarra Vega, Maikel Ayo González, Pedro Gonzalez Cabezas, Rafael Carrasco Moreno

**Affiliations:** 1 Hematology Department University Hospital Vinalopó Alicante Spain; 2 Palliative Care Unit University Hospital Vinalopó Alicante Spain; 3 University Hospital Vinalopó Alicante Spain

**Keywords:** telemedicine, digital unit, hybrid hematology department, hematology, hybrid, implementation, telehealth, monitor, monitoring, remote care, virtual care, hematological, hematologist, hematologists, service, services, delivery, holistic, digital health intervention, digital transformation

## Abstract

**Background:**

Currently, there are no telemedicine models that fully integrate all areas of hematology into daily practice.

**Objective:**

The objectives of this feasibility study were to assess the practicality of implementing telemedicine into our clinical practice in the first Digital Hematology Unit and propose an innovative integrative design for clinical practice.

**Methods:**

We designed the Digital Hematology Unit, which is a specific physical space dedicated to carrying out telemedicine and monitoring patients in a holistic way. Also, a satisfaction questionnaire was performed and health care indicators were measured.

**Results:**

In 2021, there were 1331 first visits and 7534 follow-up visits. Of the first visits, 12.2% (n=163) were face-to-face and 87.8% (n=1168) were telematic. For follow-up visits, 29.9% (n=2251) were face-to-face and 70.1% (n=5283) were telematic. The health care management indicators showed that we had a waiting time of less than 4 days and took less than 4 hours to answer interconsultations among specialists. Moreover, patients reported a high level of satisfaction with the services provided.

**Conclusions:**

Our Digital Hematology Unit, as a case of success, serves as an example of how innovative digital solutions can contribute to the quality of care and excellence in health care achieved through a digital transformation process led by hematologists.

## Introduction

Telemedicine is a rapidly evolving field that harnesses technology to remotely assess, diagnose, monitor, treat, and educate patients, with the goal of enhancing health care delivery [1]. It seems that telemedicine has a high effectiveness, particularly in managing chronic diseases, contributing to patient-centered care, and improving access to health care services [2].

Patient-centered care is associated with higher satisfaction and better health outcomes, and telemedicine can be a useful tool in achieving patient-centered models and encouraging a multidisciplinary approach [3].

In this feasibility study, we focused on the integration of telemedicine into the specialized field of hematology and hemotherapy because hematology encompasses a wide range of conditions, making its incorporation into telemedicine practice particularly challenging.

This study aimed to evaluate the feasibility of our innovative telemedicine model within the field of hematology. We present the results from our Digital Hematology Unit (DHU) in a Hybrid Hematology Department and propose it as a potential solution for the broader implementation of telemedicine in clinical hematology practice.

## Methods

### Study Design

We conducted a comprehensive evaluation of data collected during 2021 comparing face-to-face visits with telematic consultations across various hematology areas. This evaluation followed the launch of the DHU at our university hospital. The timetable included the launching of the DHU, writing of all the protocols for the use of telemedicine following societies’ guidelines in this field [4], educating patients, and finally, measuring the clinical indicators and analyzing the results of patient questionnaires. The DHU provided dedicated physical spaces for hematologists to conduct telemedicine. The infrastructure of the DHU consisted of a dedicated space solely for telemedicine patient visits shared by hematologists. It included computers, headphones, telephones, and a suitable environment for conducting video calls. Additionally, there was a screen that allowed the health care staff a view of the ward to provide a quick overview of the patients on the ward while in meetings during the morning and at the end of the day. This enabled doctors, nurses, and support staff to prioritize care, make informed decisions, and ensure patient safety and well-being. It also helped streamline communication and coordination among the health care team members and enhanced the efficiency of patient care in the DHU. In managing the DHU, it was crucial to maintain detailed records of computer equipment and resources, ensuring easy management and swift replacements when needed. Continuous improvement was also key. To this end, we gathered feedback from doctors to make enhancements based on their suggestions. Also, it was important to stay technologically updated and consider periodic upgrades to ensure optimal performance of the devices. Regarding telemedicine, we established clear protocols to safeguard patient privacy and confidentiality during consultations and established the selection criteria of patients that were suitable for telemedicine follow-up. It was also very important to provide equitable access to telemedicine services and offer training to both doctors and patients on effective telemedicine tool usage. Health care units were also established to facilitate interprofessional interaction, such as the Hematology-Home Hospitalization Unit (HHD) and the Hematology-Primary Care Unit (HAP). These units catered to inpatient treatment, palliative care, and patient assistance at home, as well as enhancing communication and collaboration with primary care. The HDD and HAP units interacted with the DHU and either the HAP care team or the HHD care team established the need for telematic follow-up of those patients in the DHU.

The DHU covered various hematology areas, including hemostasis and thrombosis, general hematology, erythropathology, oncohematology, and hemotherapy.

We conducted a patient satisfaction survey during visits to assess their experience and satisfaction levels with the different types of consultations. We also measured health care quality indicators, such as the hematological consultation response time, which evaluates how long a patient waits for medical attention, either in the waiting room or to secure an appointment.

### Ethical Considerations

This study has undergone ethical review by the Ethics Committee of Vinalopó University Hospital and was approved under reference number VP103-10/1/2021. All research activities involving human patients in this study have been treated in accordance with the ethical guidelines established by The Organic Law on Data Protection. All necessary approvals have been obtained, including for the analysis of research data.

To protect the privacy and confidentiality of the participants, all data collected in this study were anonymized before the analysis. Measures have been taken to ensure that the identifiable details of the participants are not disclosed. Informed consent was obtained, and no compensation was provided.

This study complies with the ethical provisions outlined in the informed consent, and any additional analysis is conducted in accordance with existing ethical approvals.

## Results

In 2021, our Hematology and Hemotherapy Unit conducted a total of 8865 consultations (first visits: n=1331; follow-up visits: n=7534), with 12.2% (n=163) of first visits being face-to-face and 87.8% (n=1168) being telematic. For follow-up visits, 29.9% (n=2251) were face-to-face and 70.1% (n=5283) were telematic.

Specifically focusing on consultations for oncohematology patients (n=1200), including those enrolled in specialized units, such as the Multiple Myeloma Unit (n=500) or the Lymphoma Unit (n=400), all initial consultations (n=323) were conducted face-to-face. Subsequently, during follow-up sessions, a notable shift occurred, with 53% (n=265) of consultations in the Multiple Myeloma Unit taking place via telemedicine, while 47% (n=235) remained face-to-face. For follow-up visits in the Lymphoma Unit (n=700), 75% (n=525) were face-to-face and 25% (n=175) were telematic.

Additionally, in these specialized units, specifically the Hemostasis and Thrombosis Unit, and in the responsible Blood Therapy Unit, all consultations were exclusively facilitated through telemedicine, demonstrating a complete reliance on digital platforms for health care delivery. Of the 3200 total consultations in the Hemostasis and Thrombosis Unit, 31.5% (n=1008) were telematic first visits and 68.5% (n= 2192) were telematic follow-up visits.

Furthermore, within the framework of the HAP, a total of 2122 telemedicine interconsultations transpired between general practitioners and hematologists. This initiative resulted in a substantial 20% (n=1223) reduction in face-to-face visits compared with the previous year when all consultations had been conducted in person, highlighting a significant shift toward telemedical interactions. These statistics unequivocally illustrate the successful integration and widespread acceptance of telemedicine, particularly in specialized oncological hematology units, substantiating its efficacy in transforming traditional health care models.

We have successfully achieved key health care management indicators, notably reducing visit delays from an average of 56 (SD 4) days to less than 3 (SD 1) days. Additionally, our response time for interconsultations significantly improved, with inquiries now being addressed in less than 4 hours, compared to the previous waiting period of over 24 hours. Furthermore, our telemedicine services have garnered exceptionally high patient satisfaction rates, with no reported health complaints. The results from our patient satisfaction questionnaire revealed that, of the 400 patients who completed the questionnaire, an overwhelming majority expressed satisfaction with the quality of care provided through telemedicine, with more than 90% (n=360) responding positively (Table 1).

**Table 1:** Patient satisfaction questionnaire results (n=400).

Question, n (%)	Yes	No	Don’t know	It can be improved
1. Are you satisfied with the quality of medical care provided through telemedicine in hematology?	387 (96.8)	5 (1.3)	0 (0)	8 (2)
2. Do you consider the communication with your hematologist during telemedicine consultations to have been effective?	385 (96.3)	0 (0)	0 (0)	15 (3.7)
3. Have you experienced any technical or connectivity issues during your telemedicine appointments in hematology?	21 (5.2)	355 (88.8)	12 (3)	12 (3)
4. Do you feel that telemedicine in hematology has improved your access to health care compared to in-person appointments?	355 (88.8)	12 (3)	12 (3)	21(5.2)
5. Would you recommend telemedicine in hematology to others based on your personal experience?	390 (97.6)	5 (1.2)	5 (1.2)	0 (0)

## Discussion

### Principal Results

The successful implementation of our DHU has unequivocally showcased the feasibility and effectiveness of telemedicine in hematology practice. Through the reduction of visit delays and the augmentation of both patient and physician satisfaction, we have not merely elevated health care access and quality, but have fundamentally reshaped health care management paradigms. The insights gleaned from our patient questionnaire indicated an overwhelmingly positive perception of telemedicine in hematology. One limitation stems from the fact that the survey was administered by health care staff, introducing biases into the collection process.

The process of modernizing hematology services through digital transformation and establishing a comprehensive care platform for hematology patients in an eHealth environment presents a daunting challenge. However, our successful implementation of a DHU has paved the way for achieving outstanding health care management indicators. We have achieved exceptionally high levels of patient satisfaction. By enhancing access to care, reducing wait times, and improving the overall patient experience, we are not only enhancing people’s health and well-being but also revolutionizing health care management models. Furthermore, our efforts have led to a reduction in in-person visits, contributing to the sustainability of the health care system. There have already been some success stories in the field of hematology, particularly in malignant hematology, where telehealth interventions have been effective in reducing face-to-face consultations. Numerous such studies have reported positive results [5-11], however, most of these studies are retrospective, descriptive, and do not specifically focus on all hematological patients [12-19].

In the realm of hemostasis and thrombosis, telemedicine has demonstrated a significant positive impact on anticoagulation [20-31]. During the COVID-19 pandemic, telemedicine platforms were effectively used to monitor patients with different hematological diseases [32-35]. Despite initial skepticism and potential barriers, our digital unit has achieved remarkable success, boasting high patient satisfaction rates. We have recently taken a step forward by implementing disruptive technologies, such as artificial intelligence, app-based tracking for nutrition and exercise, and a virtual assistant for patient follow-up, which have all contributed to improving the patient experience and overall quality of care.

### Conclusion

In summary, our study underscores the potential of telemedicine in enhancing hematology practice, making it more patient-centered, accessible, and efficient. Our success story serves as a beacon, demonstrating how innovative digital solutions can significantly contribute to the quality of care and excellence in health care.

## Abbreviations

DHU: Digital Hematology Unit
HDD: Hematology-Home Hospitalization Unit
HAP: Hematology-Primary Care Unit

## Data Availability

Our data adheres to open science availability guidelines for broader research accessibility.
